# Tooth Resorption – Part 1: The evolvement, rationales and controversies of tooth resorption

**DOI:** 10.1111/edt.12757

**Published:** 2022-05-12

**Authors:** Shaul Lin, Daniel Moreinos, Arieh Y. Kaufman, Paul V. Abbott

**Affiliations:** ^1^ Department of Endodontic and Dental Trauma Rambam Health Care Campus Haifa Israel; ^2^ The Ruth and Bruce Rappaport Faculty of Medicine Technion ‐ Israel Institute of Technology Haifa Israel; ^3^ The Gertner Institute Emergency Management and Disaster Medicine Sheba Medical Center Tel Hashomer Israel; ^4^ Endodontics Department Galilee Medical Center Nahariya Israel; ^5^ The Azrieli Faculty of Medicine Bar‐Ilan University Safed Israel; ^6^ Department of Endodontology The Maurice and Gabriela Goldschleger School of Dental Medicine, Tel Aviv , University, Tel Aviv Israel; ^7^ UWA Dental School The University of Western Australia Perth Western Australia Australia

**Keywords:** dental trauma, inflammatory root resorption, replacement resorption, surface resorption

## Abstract

In 1966, Andreasen and Hjørting‐Hansen were the first to describe a relationship between tooth resorption and dental trauma. However, Andreasen's original classification did not include other resorptive processes which have since been identified. Numerous articles have been published suggesting new terminology and definitions for tooth resorption. A uniform language with universally accepted terminology is crucial to eliminate the multiplicity of terms and definitions which only cause confusion within the profession. An electronic literature search was carried out in the PubMed database using the following keywords for articles published in English: “root resorption,” “inflammatory root resorption,” “replacement resorption,” “cervical resorption,” “trauma,” “ankylosis,” “surface resorption,” and “internal resorption.” The search also included textbooks and glossaries that may not have surfaced in the online search. This was done to identify articles related to tooth resorption and its etiology in dentistry. The aim of this review was to present the history that has led to the variety of terms and definitions for resorption. This review emphasizes the need for a clearer, simpler, and more comprehensive nomenclature for the various types of tooth resorption which are presented in Part 2 of this series.

## 1 INTRODUCTION

Resorption is defined as either a physiological or a pathological process which results in loss of substance from a tissue. In the case of tooth resorption, the outcome of the process is the loss of dentin and/or cementum.^1^ Some types of tooth resorption are also associated with resorption of the adjacent bone along with loss of the periodontal ligament (PDL).^1^ Historically, evidence of tooth resorption was first described in the literature at the beginning of the 20th century.^2^ In his paper in 1901, Miller described what later became to be known as external cervical (invasive) resorption, and he hypothesized that such resorption was due to infection within the root canal system^2^ but that hypothesis has not been proven. In 1966, Andreasen and Hjørting‐Hansen were the first to describe pathological tooth resorption based on clinical, radiographic, and histologic findings in teeth after trauma.^3^, ^4^ They reported three different pathological responses of the tooth, PDL, and bone subsequent to trauma and a fourth response with no resorption. They named these responses as inflammatory resorption, replacement resorption, surface resorption, and healing with no resorption. Inflammatory resorption and replacement resorption were observed radiographically.^3^ Later, in a histological evaluation of avulsed teeth, surface resorption was identified histologically but could not be detected radiographically.^4^ Two other types of pathologic resorption were later added—first by Andreasen in 1975^5^ and the second by Andreasen in 1986.^6^ These were called transient replacement resorption and transient apical breakdown, respectively.^5^, ^6^ The latter is a condition where there is resorption of both bone and tooth substance as the root undergoes a remodeling process. It is no longer considered to be a pathological process but instead is considered to be part of a repair process that occurs in some cases. Hence, it will not be discussed further in this paper.

Inflammatory resorption (currently termed external inflammatory resorption) is radiographically characterized by the loss of tooth substance (cementum, dentin, and PDL) with an adjacent radiolucency in the bone.^7^ It has been reported that inflammatory resorption can be arrested following adequate root canal treatment and later radiographic follow‐up examination usually shows repair of the resorptive defects with re‐establishment of normal PDL space.^7^ In cases where endodontic treatment was not performed, the inflammatory resorption extended markedly, the teeth developed periapical radiolucencies indicating apical periodontitis as a result of the infected root canal system, and most often the outcome was loss of the tooth within the first year following trauma.^3^ The term “inflammatory resorption” was adopted by most researchers and the American Association of Endodontists (AAE) in their glossary to describe not only teeth with this resorption after trauma but also teeth with apical resorption and a periapical radiolucency due to an infected root canal system.^1^, ^8^, ^9^

**FIGURE 1:**
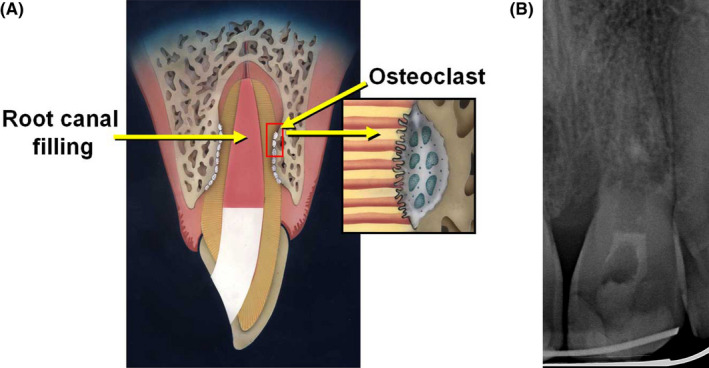
(A) Diagrammatic representation of ankylosis with external replacement resorption (reproduced with permission from Fuss *et al*.^21^). (B) Radiographically, ankylosis is recognized by the lack of lamina dura and PDL while external replacement resorption is characterized by loos of tooth structure which is replaced by bone. This radiograph demonstrates a case of extensive ankylosis and replacement resorption of tooth 21 18 months after the tooth had been avulsed, kept dry for 2 h and then replanted

Replacement resorption is the second type of response described by Andreasen and Hjørting‐Hansen.^3^, ^4^ It is characterized by a pathologic loss of tooth substance (cementum, dentin, and PDL) with subsequent replacement of these tissues by bone, which results in fusion of the root to the surrounding bone.^1^ This type of resorption occurs following severe trauma to teeth—such as intrusion, lateral luxation or avulsion—where the PDL and a large portion of the root surface have been damaged (typically, more than 20% of the root surface).^7^ The initial process is known as ankylosis which is defined as loss of the PDL.^4^ Ankylosis is effectively a fusion of the alveolar bone and dentin. It occurs when osteoclasts from the adjacent alveolar bone evolve at an early stage after the injury and adhere to the damaged root surface before the fibroblasts can reach the area to repair the PDL.^4^, ^7^ Once ankylosis occurs, replacement resorption will follow as tooth substance is resorbed by clastic cells and then it is replaced by bone.^4^, ^7^ Ankylosis is recognized radiographically by the lack of lamina dura and PDL (Figure 1), and clinically it is characterized by a metallic, higher pitched, or different sound when percussing the tooth. The term “replacement resorption” was also adopted by most researchers and the AAE in their Glossary.^1^, ^10^, ^11^ Unlike inflammatory resorption, replacement resorption usually cannot be treated effectively, although occasionally it may be transient if only a small area of the root is involved.^5^ Recently, Yoshpe et al.^12^ presented a series of cases in which traumatized teeth developed ankylosis and regenerative endodontic treatment succeeded in arresting the advancement of the resorptive process and even reversed it.^12^ However, more research is required to investigate this treatment in the case of ankylosis.

Surface resorption is a type of resorption of an inflammatory nature which is caused by an injury to the root surface.^3^, ^4^ The inflammation can be caused by mechanical stimuli (such as trauma or pressure) or by bacteria on the root surface.^3^ If the stimulus is only present for a short period of time, healing may take place without the need for intervention. In most cases, the resorptive defects are repaired with the formation of cementoid‐like tissue.^13^ If the affected area is larger and expands into the dentin, new cementum will follow, thus the contour of the root surface may only be partially restored.^3^, ^4^, ^8^, ^13^ This type of healing usually takes about 4 weeks to complete.^14^, ^15^ The type of the tissue that covers the resorbed root surface is dependent on the size of the area of root damage and the relative proximity of the cells to the damaged root surface.^4^ It is also dependent on how far and how fast the cells travel in order to cover the damaged root surface. A localized injury over a small surface area favors cemental healing which is not detectable on radiographs (Table 1).^4^ The complete healing of a tooth is characterized histologically by regeneration of the PDL with no radiographic signs of tooth or bone resorption.^3^, ^4^, ^6^

**TABLE 1:** Tooth resorption ‐ terms and mechanisms

Author and year/article type	Nomenclature		Properties	Quote
Andreasen and Hjørting‐Hansen 1966^3^ (Retrospective)		Surface resorption	The resorption was observed radiographically, clinically and histologically in teeth after trauma	*Surface resorption* could be identified only histologically
Andreasen and Hjørting‐Hansen 1966^4^ (Retrospective)		Inflammatory resorption		
		Replacement resorption		
Andreasen 1970^30^ (Retrospective)	External	Inflammatory root resorption	The resorption was observed radiographically and clinically, in teeth after trauma	Root resorption is presumably first of all related to the degree of trauma inflicted to the periodontium. Inflammatory root resorption and replacement resorption both considered progressive root resorption
		Replacement resorption		
		Surface resorption		
	Internal	Internal root resorption		
		Internal replacement Resorption		
Andreasen 1975^5^ (Retrospective)	External	Transient replacement resorption	The resorption was observed radiographically and clinically, in teeth after trauma	*Transient replacement resorption* decreased mobility values indicate that ankylosis was usually evident 5 weeks after replantation. At the same time, it was possible to diagnose the ankylosis by the percussion test. The radiographic examination first revealed ankylosis 8 weeks after replantation
Andreasen 1985^13^ (Review)	External	Surface resorption	Resorption in relation to dental traumatology, pedodontics, periodontics, orthodontics and endodontics, based on Andreasen's (1966) nomenclature	*Surface resorption*, deep cavities penetrating into dentinal tubules and communicating with infected, necrotic pulpal tissue or a pus zone can change into inflammatory resorption cavities. Can be observed also radiographically
		Inflammatory resorption		
		Replacement resorption		
Andreasen 1986^5^ (Retrospective)	External	Transient apical breakdown	The resorption was observed radiographically in teeth after trauma	*Transient apical breakdown* periapical pathology after trauma, has been assumed not to disappear spontaneously. During evaluation of a larger sample of luxated permanent teeth, however, it became evident that transient apical breakdown (TAB) could occur; that is, normalization of apical conditions without intervening treatment
Feiglin 1986^16^ (Review)	External	Physiological resorption Orthodontic tooth resorption Pressure from adjacent tooth or cyst		The primary dentition undergoes gradual loss of its root
Tronstad 1988^8^ (Review)	Transient root resorption External/Internal	Transient inflammatory resorption	The resorption is based on inflammatory reaction of teeth after trauma	*Transient inflammatory resorption* may occur on the root apices of non‐traumatized teeth with infected necrotic pulps Transient root resorption as such is without clinical importance and the resorption defects are usually too small to even be detected radiographically (Also known as surface resorption)
		Transient internal inflammatory resorption		
		Progressive external inflammatory resorption		*Progressive external inflammatory* resorption, orthodontic or endodontic problem. External inflammatory resorption may occur on the root apices of non‐traumatized teeth with infected necrotic pulps A radiograph will reveal a periapical radiolucency associated with shortened root apices with a roughened root end
	Progressive external inflammatory resorption is sub‐divided into three groups:	Cervical resorption		
		Dento‐alveolar ankylosis and replacement resorption		*Dento‐alveolar ankylosis* clinically, is recognized because of a lack of mobility of ankylosed teeth. These teeth will also have a special metallic percussion sound, and after some time they will be in infra‐occlusion. Radiographically, dento‐alveolar ankylosis may be recognized by the absence of a PDL space
		Progressive internal resorption		*Progressive internal resorption* is the resorptive activity sustained by infection of necrotic pulp tissue in the root canal coronally to the area where the resorption takes place
Trope 1998^9^ (Review)	External Root Resorption Type 1—Attachment Damage Alone	Attachment Damage Alone: Pressure, Mild and Severe Traumatic Injury (Ankylosis)	Resorption based on etiology, in relation to dental traumatology, pedodontics, periodontics, orthodontics and endodontics	

Type 2—Infection Alone	Apical periodontitis, marginal periodontitis		

Type 3—Attachment Damage Plus Infection	Peri‐radicular root resorption of pulpal origin, Sub‐attachment root resorption of sulcular origin		*Periradicular root resorption* of pulpal origin, Sub‐attachment root resorption of sulcular origin caused by a traumatic injury and the inflammatory stimulator is infection (pulpal space or sulcus)
	Internal root resorption	Internal root resorption	
Ne et al. 1999^33^ (Review)	Internal resorption	Internal replacement resorption		*Internal replacement resorption*. Root canal replacement resorption involves resorption of the dentin and a subsequent deposition of hard tissue that resembles bone or cementum, but not dentin. This type of resorption takes place when a chronic inflammatory process occurs juxtaposed to a region in which the odontoblastic layer and pre‐dentine are absent or damaged, which can occur as a result of trauma or application of extreme heat to the tooth
	External resorption	External surface resorption		
		Replacement resorption		
		Ankylosis		
		External inflammatory root resorption		*External resorption* Further categorized into cervical resorption with or without a vital pulp (invasive cervical root resorption) and external apical root resorption. Other variations of resorption include combined internal and external resorption and transient apical breakdown
Trope 2002^22^ (Review)	I. External resorption	a) Stimulus of short duration (transient stimulus)	Diagnosis is based on etiology, in relation to dental traumatology, pedodontics, periodontics, orthodontics and endodontics	
		b) Stimulus of long time periods (progressive stimulus): pressure and pulp space infection (*apical* and *lateral periodontitis*)		
		c) Sulcular infection		
	II. Internal resorption	Internal resorption		
Fuss et al. 2003^29^ (Review)		I. Pulpal infection root resorption	Resorption is according to stimulation factors, in relation to the different dentistry disciplines	*Pulpal infection root resorption ‐* the most common stimulation factor for root resorption is pulpal infection following injury to the precementum or predentin, infected dentinal tubules, may stimulate the inflammatory process with osteoclastic activity in the periradicular tissues or in pulpal tissues
		II. Periodontal infection root resorption		*Periodontal infection root resorption* may occur after injury of the precementum, apical to the epithelial attachment, followed by bacterial stimulation originating from the periodontal sulcus. Injury may be caused by dental trauma, and chemical irritation maybe caused by hydrogen peroxide 30%, orthodontic treatment, or periodontal procedures
		III. Orthodontic pressure root resorption		
		IV. Impacted tooth or tumor pressure root resorption		
		V. Ankylotic root resorption		
		Internal inflammatory resorption	Resorption based on clinical and histological manifestations, in relation to the different disciplines of dentistry (dental traumatology, pedodontics, periodontics, orthodontics and endodontics)	
Heithersay 2007^31^ (Review)	Trauma induced tooth resorption	Surface resorption	Resorption based on etiology and dynamics in relation to dental traumatology, pedodontics, periodontics, orthodontics and endodontics	

Transient apical internal resorption			*Transient apical internal resorption* is another form of trauma induced non‐infective root resorption

Pressure resorption and orthodontic resorption		*Pressure resorption and orthodontic resorption*, Root resorption following orthodontic treatment, with the removal of the initiating “trauma”, these non‐infective resorptions will become inactive and uncomplicated repair will occur

Replacement resorption		

Infection‐induced dental resorption	Apical internal inflammatory (infective) root resorption		

	Intra‐radicular internal inflammatory (infective) root resorption		

External inflammatory root resorption		External inflammatory root resorption this type of external root resorption occurs when infection is superimposed on a traumatic injury. Nevertheless it can also be induced in some cases of endodontic pathosis as peri‐radicular infection

Communicating internal‐external inflammatory resorption		*Communicating internal‐external inflammatory resorption* Where resorption has extended from an internal inflammatory resorption to involve the external surface a communicating lesion is created

Hyperplastic invasive resorptions	Internal replacement (invasive) resorption		*Internal replacement (invasive) resorption*. This type of resorption is relatively rare and may appear clinically as a pink area in the crown of the affected tooth. While this may be internal in origin, particularly if there has been a history of recent trauma, it more commonly arises from an external periodontal source. The location of the pink spot is more likely to be entirely within the crown of the tooth in internal replacement (invasive) resorption

Invasive cervical resorption		*Invasive cervical resorption* characterized by its cervical location and invasive nature, In the absence of treatment, invasive cervical resorption leads to destruction of tooth structure. Resorption of coronal dentine and enamel often creates a clinically obvious pinkish colour in the tooth crown as highly vascular resorptive tissue becomes visible through thin residual enamel
Patel & Pitt Ford 2007^34^ (Review)	External resorption	Surface resorption	Resorption in relation to dental traumatology, pedodontics, periodontics, orthodontics and endodontics, based on of Andreasen's (1966)^3^ and the AAE^1^ nomenclature	
		External inflammatory resorption		
		External replacement resorption		
		External cervical resorption		
		Transient apical breakdown		
	Internal resorption	Internal inflammatory resorption		
AAE Glossary of Endodontic Terms (Official nomenclatures ‐ Glossary)^**1**^	External resorption	External surface resorption	Resorption in relation to dental traumatology, pedodontics, periodontics, orthodontics and endodontics, based on Andreasen's (1966)^3^ nomenclature	*External surface resorption. A physiologic process causing small superficial defects in the cementum and underlying dentin that undergo repair by deposition of new cementum*
		External inflammatory resorption		*External inflammatory resorption*. An internal or external pathologic loss of tooth structure and possibly bone, resulting in a defect that occurs as the result of microbial infection; characterized radiographically by radiolucent areas along the root
		External cervical resorption		*External cervical resorption*. A type of external resorption that occurs in the coronal third of the root
		External replacement resorption		*External replacement resorption*. A pathologic loss of cementum, dentin and PDL with subsequent replacement of such structures by bone, resulting in fusion of bone and tooth
		Transient apical breakdown		*Transient apical breakdown*. Response to tooth luxation consisting of radiographic apical bone and root resorption that resolves spontaneously without intervening treatment
	Internal resorption	Internal inflammatory resorption		*Internal inflammatory resorption*. An inflammatory process initiated within the pulp space with loss of dentin and possible invasion of the cementum
Darcey & Qualtrough 2013^35^ (Review)		External surface resorption	Resorption in relation to the different disciplines of dentistry (dental traumatology, pedodontics, periodontics, orthodontics and endodontics)	*Surface resorption* is usually sub‐clinical. Radiographically it may be seen as cavitation in the cementum and dentine, or an alteration of the root contour
	External inflammatory resorption	Sterile inflammatory resorption		*Sterile inflammatory resorption* an acute inflammatory process and the tooth becomes tender to pressure or there is an associated swelling or the reduced support leads to mobility. The resorptive lesion is often an incidental radiographic finding. Presentation will vary depending upon whether the process is infective or sterile
		Infective inflammatory resorption		
		External cervical resorption		
		External replacement resorption		
		Internal inflammatory resorption		
		Internal replacement resorption		
Patel & Saberi 2018^36^ (Review)	External	External inflammatory resorption	Resorption based on etiology and dynamics in relation to dental traumatology, pedodontics, periodontics, orthodontics. and endodontics, based on the nomenclature of Andreasen (1966)^3^	
		External replacement resorption		
		External cervical resorption		
		External surface resorption		*External Surface resorption* This is a non‐infective, transient, pressure‐induced resorption. This resorptive process will stop progressing once the source of the pressure has been removed, resulting in repair of the resorbed root‐face with cementum. Orthodontic tooth movement, impacted teeth, tumors and cysts have been reported to cause surface resorption
		Transient apical breakdown		
	Internal	Internal inflammatory resorption		
		Internal replacement resorption		*Internal replacement resorption* Histological appearance of the defects reveals the presence of metaplastic hard tissue replacing the resorbed dentine at the periphery of the defect, which is suggestive of active and simultaneous resorption and replacement. This metaplastic hard tissue resembles cementum or osteoid‐like tissues. However, detailed pathogenesis is not fully understood. It has been suggested that IRR is an attempt of IIR to replace the damaged (resorbed) dentine. Diagnosis is made radiographically

In 1975, Andreasen reported the outcomes for 35 patients who had 40 permanent teeth avulsed and replanted.^5^ In this report, a new condition, namely “transient replacement resorption,” was used to describe teeth that initially had reduced mobility values which later returned to normal. Radiographic signs of transient replacement resorption were present in some cases. The teeth that exhibited transient replacement resorption had a significantly shorter extra‐oral dry period in comparison with teeth diagnosed with progressive replacement resorption. Later this type of response became known as surface resorption.^13^ As outlined above, this type of resorption is an inflammatory process in response to a stimulus caused by damage to the root surface as a result of the injury. Without any further stimuli, this process will usually be self‐limiting and healing will occur without any intervention. In most instances, the resorptive defects are repaired with the formation of cementoid‐like tissue.^3^, ^5^

The definition of root resorption subsequent to dental trauma, described by Andreasen and Hjørting‐Hansen,^3^, ^4^ was adapted by Feiglin in 1986^16^ and Tronstad in 1988^8^ to cover additional clinical situations. Tronstad based his nomenclature on the concept that all resorptive reactions are caused by inflammation which can also occur under sterile conditions (such as after concussion or orthodontic tooth movement) or the resorption may be associated with infection (currently termed external inflammatory resorption).^8^ He then sub‐divided inflammatory resorption into transient and progressive resorption, which could be internal, external, or cervical (although cervical resorption is no longer considered to be inflammatory or transient). This kind of resorption requires mineralization of, or damage to, the protective soft tissue layer (predentin or precementum). A long‐lasting stimulus, such as a sharp edge, pressure, infection, or various systemic diseases (for example, a cyst, ameloblastoma, giant cell tumor, fibro‐osseous dysplasia) will lead to progressive inflammatory resorption (today, this resorption is termed either surface or pressure resorption). Removal of the stimuli in most cases will lead to cessation of the resorption and healing with a cementum‐like tissue, and thus, it can be denoted as a transient resorptive process. Without proper treatment, namely removing the stimuli, the resorption will continue and might lead to continued destruction of the tooth, which is known as progressive resorption.^8^

Cervical resorption (known today as external cervical resorption, external invasive resorption, and various other names—see Tables 1 and 2) was described by Harrington and Natkin in 1979 as a resorptive process related to bleaching of pulpless teeth after trauma.^17^ Additional reports supported the etiologies of trauma with bleaching or bleaching alone as the cause(s) of this invasive resorption due to changes in the composition of the marginal tissue which make it less resistant to resorption.^18^, ^19^, ^20^ Tronstad^8^ offered a different theory which was based on the theory proposed by Cvek and Lindvall in 1985,^21^ whereby injury to the cervical attachment apparatus can lead to a resorption lacuna. The theory proposed then was that if stimuli from bacteria originating in the gingival sulcus exist, the resorption will continue and will penetrate into the dentin but it will usually only affect the dental pulp in its later stages since the predentin protects the pulp from the resorbing cells.^8^ When the resorption is long‐standing, invading tissue may be seen undermining the enamel of the crown of the tooth, resulting in a pink appearance of the tooth.^22^ However, to date, there is still great controversy among researchers and clinicians regarding the etiology, the nature of this type of resorption, and the terminology used. This type of resorption differs from inflammatory root resorption in that it frequently occurs in teeth with normal, healthy pulps and it is not associated with bacteria. Pulp involvement is rare and only occurs in severe or advanced cases.^23^ Although the etiology remains vague, potential predisposing factors for this resorptive process include trauma, orthodontics, periodontal therapy, and internal bleaching.^24^ Mavridou et al.^25^ have reported that most of their external cervical resorption cases were observed in maxillary teeth (72%). The most frequent factor was orthodontics (45.7%) followed by trauma (28.5%), parafunctional habits (23.2%), poor oral health (22.9%), malocclusion (17.5%), and extraction of a neighboring tooth (14%).^25^ The use of nano‐CT, histology, and 3‐dimensional imaging by Mavridou et al. has significantly improved the understanding of the histopathology of this type of resorption.^26^, ^27^

**TABLE 2:** Different terms describing the same type of tooth resorption

AAE Glossary^a^	Analogous terminology
External surface resorption	Surface resorption—Andreasen & Hjørting‐Hansen 1966^4^ Transient inflammatory resorption—Tronstad 1988^8^ Periapical replacement resorption—Bender 1997^28^ Pressure resorption and orthodontic resorption—Patel & Pitt Ford 2007^34^ Orthodontic‐induced external root resorption—Alhadainy et al. 2019^37^
External inflammatory resorption	Pulpal infection root resorption—Fuss et al. 2003^29^ Progressive inflammatory resorption—Tronstad 1988^8^ External inflammatory resorption—Patel & Pitt Ford 2007^34^ Peripheral inflammatory root resorption—Ne et al. 1999^33^ External inflammatory root resorption—Heithersay 2007^31^ Inflammatory resorption—Andreasen & Hjørting‐Hansen 1966^3^ Peri‐radicular root resorption of pulpal origin—Trope 1998^9^
External replacement resorption	Replacement resorption—Andreasen & Hjørting‐Hansen 1966^3^ Ankylosis root resorption—Fuss et al. 2003^29^ Ankylosis—*Ne* et al. 1999^33^ Replacement resorption—Ne et al. 1999^33^ Gunraj 1999^11^ Dento‐alveolar ankylosis and replacement resorption—Tronstad 1988^8^
External cervical resorption	Periodontal infection root resorption—Fuss et al. 2003^29^ Invasive cervical resorption—Heithersay 1999^24^ Extra‐canal invasive resorption—Frank 1998^23^ Sulcular infection—Trope 1998^9^ Peripheral inflammatory root resorption—Ne et al. 1999^33^ Cervical resorption—Tronstad 1988^8^ Gunraj 1999^11^ Cervical inflammatory root resorption—Feiglin 1986^16^ Mid‐root and cervical external resorption—Gartner 1976^38^
Internal inflammatory resorption	Progressive internal inflammatory resorption—Tronstad 1988^8^ Intra‐radicular internal resorption—Heithersay 2007^31^ Radial pulp enlargement resorption—Kanas & Kanas 2011^48^
Transient apical breakdown	Transient apical breakdown—Andreasen 1986^6^
Others	Transient internal resorption—Tronstad 1988^8^ Internal replacement resorption—Andreasen 1970,^30^ Ne et al. 1999,^33^ Heithersay 2007^31^ Transient replacement resorption—Andreasen 1975^5^

^a^ AAE Glossary.

Initially, surface resorption was considered to not be identifiable radiographically but only histologically.^2^ In 1985, Andreasen named asymptomatic apical resorption following orthodontic treatment as “external surface resorption.”^13^ A number of terms have been used to identify the resorption of roots that has been induced by orthodontic tooth movement—such as “orthodontic tooth resorption” by Feiglin^16^ and “transient inflammatory resorption” by Tronstad^8^ (Table 2). In 1997, Bender et al.^28^ proposed a new term for orthodontic resorption, namely “periapical root resorption”. This type of resorption occurs at the apical end of teeth undergoing orthodontic treatment and most commonly occurs in incisors. Interestingly, this type of resorption occurs significantly less often in endodontically treated teeth undergoing orthodontic treatment.^28^

Various other terms and classification systems have been used in the literature for the different types of resorption. In 2003, Fuss et al.^7^ based their terminology and definitions of resorption on the source of the stimulating factors, which include pulp infection resorption, periodontal infection resorption, orthodontic pressure resorption, impacted tooth or tumor pressure resorption, and ankylotic resorption. Removal of the stimulating factor (ie, pressure, orthodontic appliances, pulp infection, or periodontal infection) is a crucial part of the treatment of these types of resorption.^7^ Based on the nomenclature in the AAE Glossary and Andreasen's terms in 1970 and 1975,^1^, ^5^, ^29^ Heithersay^30^ proposed an alternative classification of tooth resorption that was adopted by Lindskog et al.^31^ This classification sub‐divided resorption into the following three broad groups: (1) trauma‐induced tooth resorption; (2) infection‐induced tooth resorption; and (3) hyperplastic invasive tooth resorption. However, there are several problems with this classification. The main one is that it is not possible to assign some types of resorption to just one of these groups. For example, one could argue that external inflammatory resorption which is included in the “infection‐induced resorption” group could also be included in the “trauma‐induced resorption” group. Furthermore, pressure resorption and orthodontic resorption are not “trauma‐induced” since trauma is usually considered to be an injury that is accidental in nature. There are also inconsistencies in the style of terminology used for the different types of resorption listed, such as the use of locations within the tooth for some types (cervical, coronal) but not for other types of resorption. Other authors^7^, ^9^, ^22^, ^30^, ^32^, ^33^, ^34^, ^35^ have also created new terms or proposed different definitions for the various types of tooth resorption, and most of them have published reviews rather than research‐based reports. As a result, these numerous and diversified classifications and definitions are inconsistent and confusing (Table 2).

The aim of this review was to present the history that has led to miscommunication between clinicians due to the variety of terms and definitions used for tooth resorption. These findings emphasize the need for clearer, simpler, and more comprehensive nomenclature for the various types of tooth resorption.

## 2 METHOD

An electronic literature search was carried out in the PubMed database using keywords listed in the Entree Terms database for articles published in English. The search employed a combined search strategy using the keywords “root resorption,” “inflammatory root resorption,” “replacement resorption,” “cervical resorption,” “trauma,” “ankylosis,” and “surface resorption.” This was done to identify articles related to tooth resorption and its etiology in dentistry. The search also included textbooks and glossaries that may not have surfaced in the online search, which were manually identified along with relevant treatment recommendations for dentistry. Inclusion criteria included studies on humans and animals, as well as articles that included nomenclature in relation to trauma and dentistry. All titles and abstracts were screened by the lead author (SL) for studies that met the eligibility criteria. Any questionable titles were discussed by all authors and the decision to include or exclude was made accordingly once consensus was reached. These titles were manually identified along with the relevant dental treatment recommendations. Exclusion criteria were studies that failed to meet the inclusion criteria, as well as conference proceedings, lectures, and letters to editors.

## 3 REVIEW

There are numerous classifications and terms for the same types of tooth resorption. For example, “apical replacement resorption” has been used for apical root resorption following orthodontic treatment.^28^ The same pathological process has been included under the category of “inflammatory root resorption,”^8^ “superficial resorption,”^13^ “orthodontic‐induced external root resorption,” and “orthodontic tooth resorption.”^22^, ^36^ This type of resorption has been named by the AAE as external surface resorption^1^ and it includes resorption due to a tumor, an impacted tooth, etc. The same problem exists for external cervical invasive resorption where at least 13 different terms have been used in various articles for this type of resorption—namely, “external resorption,” “invasive cervical resorption,” “root resorption due to periodontal infection,” “external invasive resorption,” “extra‐canal invasive resorption,” “odontoclastoma,” “fibrous dysplasia of teeth,” “burrowing resorption,” “peripheral cervical resorption,” “cervical external resorption,” “supra‐osseous extra‐canal invasive resorption,” “peripheral inflammatory root resorption,” and “periodontal infection resorption.” However, they all refer to the same condition.^1^, ^7^, ^17^, ^24^

There have been various attempts to classify tooth resorption according to etiology,^7^ treatment,^16^ the site of the resorption,^8^, ^13^, ^37^, ^38^ or the pathological process causing the inflammation and resorption.^8^ When exploring resources of nomenclature in endodontics, such as the AAE Glossary of Endodontic Terms,^1^ the general definition of resorption is based on the general anatomic origin of the resorption in the root—that is, external or internal. This definition is vague and unclear. Definitions of specific types of resorption are also very general and somewhat vague—for example, “surface resorption” is defined as “a physiologic process causing small superficial defects in the cementum and underlying dentin.” This definition could include apical resorption due to orthodontic treatment or external apical resorption due to an adjacent pathologic process such as an ameloblastoma that can occur in teeth with normal, healthy pulps.^22^ Hence, this definition is not specific enough to enable clinicians to distinguish between the different types of resorption.

The term “replacement resorption” can be problematic since whenever tooth substance is resorbed, it can be replaced by various types of tissue. Regardless of the etiology of the resorption, the replaced tissue could be bone, granulation tissue, cementum, or cementum‐like tissues. Tronstad categorized this type of resorption under “inflammatory root resorption”.^8^ Replacement and inflammatory resorption are related to completely different etiologies, thus they require different terminology and different treatment protocols.^8^, ^10^

**FIGURE 2:**
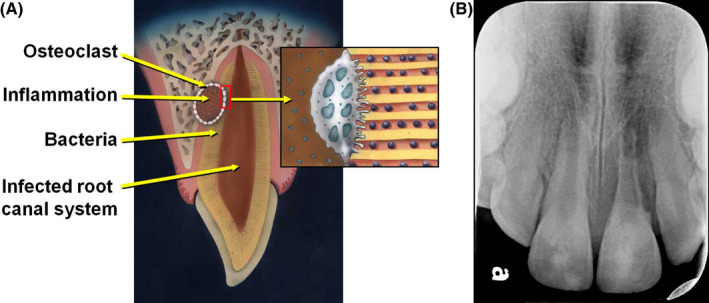
(A) Diagrammatic representation of external inflammatory resorption (reproduced with permission from Fuss *et al*.^21^). (B) Radiographically, external inflammatory resorption is characterized by radiolucencies within both the root and the adjacent bone. This radiograph demonstrates a case of very advanced and extensive external inflammatory resorption 1 year following avulsion and replantation of tooth 21. Root canal treatment was not initiated earlier because the patient did not return for the recommended regular review appointments

Similarly, inflammatory resorption is defined in the AAE Glossary of Endodontic Terms^1^ as “an internal or external pathologic loss of tooth structure and possibly bone, resulting in a defect; occurs as the result of microbial infection; characterized radiographically by radiolucent areas along the root.” This is not a synonym of the previous definition and it cannot be used to distinguish between resorption that is associated with infection alone or infection after trauma. This definition is also inaccurate as it states “loss of tooth structure and possibly bone” but bone loss does not occur with internal inflammatory resorption and it always occurs adjacent to the resorptive defect with external inflammatory resorption. The bone loss will appear as a radiolucency which is indicative of the space being occupied by the inflammatory response. This definition can include both an infected tooth with apical resorption, and teeth where the resorption is occurring anywhere along the length of the root following trauma (such as, avulsion or intrusion; Figure 2). The management of inflammatory resorption with lateral root and bone resorption following trauma is different to that for apical inflammatory resorption where there has been no previous trauma.^39^, ^40^ External inflammatory resorption can be divided into two sub‐divisions according to the etiology and the location of the resorption as described by Trope^22^ and Sak et al.^38^ External apical inflammatory resorption is an infection‐induced resorptive process which usually responds favorably to routine root canal treatment.^41^ External lateral inflammatory resorption is correlated with severe luxation or avulsion injuries. The method of choice to treat a tooth with lateral inflammatory resorption has traditionally been to medicate the root canal system with long‐term calcium hydroxide dressings until a continuous PDL space is observed radiographically along the root.^40^, ^42^, ^43^ Recent studies on treatment with regenerative endodontics^44^, ^45^, ^46^and the use of corticosteroid‐antibiotic compounds^39^, ^40^ as intracanal dressings have also been shown to be successful in arresting external lateral inflammatory resorption, and in much shorter time periods.

Endodontics is one part of the medical and dental professions that uses several terms and definitions to describe the same disease or condition. This problem exists not only in reference to tooth resorption,^47^ but also in classifications of pulp, root canal, and periapical conditions.^40^, ^43^, ^48^ This situation can lead to miscommunication between researchers, clinicians, and students resulting in a negative effect both on research and on clinical practice when treating patients. A clearer, simpler, and more comprehensive nomenclature needs to be developed for the various types of tooth resorption and this will be the subject of Part 2 of this series of articles.

## 4 CONCLUSIONS

Tooth resorption is a complication that can lead to the loss of a tooth. There are several types of tooth resorption with each having different etiology, pathogenesis, and management. There are many classifications and terms used to describe the same types of resorption which creates confusion amongst educators, researchers, authors, clinicians, and students. It can also lead to misconceptions which may eventually compromise the patient's treatment. Therefore, it is essential to develop a new clinically related and relevant classification of tooth resorption which will improve communication and provide clarity about each type of resorption.

## 5 CONFLICT OF INTEREST

The authors declare that there are no conflicts of interest in this study.

## 6 AUTHOR CONTRIBUTION

All authors contributed equally to the concept, design, literature searching, writing, revising and editing of this article.

7

## Data Availability

Data sharing is not applicable to this article as no new data were created or analyzed in this study.
